# Supplementary material for: Predicting visual function by interpreting a neuronal wiring diagram
Source: Nature. 2024 Oct 2;634(8032):113–23. doi: 10.1038/s41586-024-07953-5 (PMC11446822; doi:10.1038/s41586-024-07953-5)
Supplement: Supplementary file 6 — CRF and ERF predictions for individual TmY4 and TmY9 cells. Analogous to Supplementary Data 3, but for TmY target types. Shown are the top four monosynaptic pathways, the strongest pathway passing through each of the top ten intermediary types (ranking from Extended Data Fig. 7), and the trisynaptic pathway Tm1–TmY–Dm3–TmY (see the section entitled Prediction of spatial normalization). [file 41586_2024_7953_MOESM6_ESM.zip › DataS4/TmY9qΓèÑ/720575940630218039.pdf]

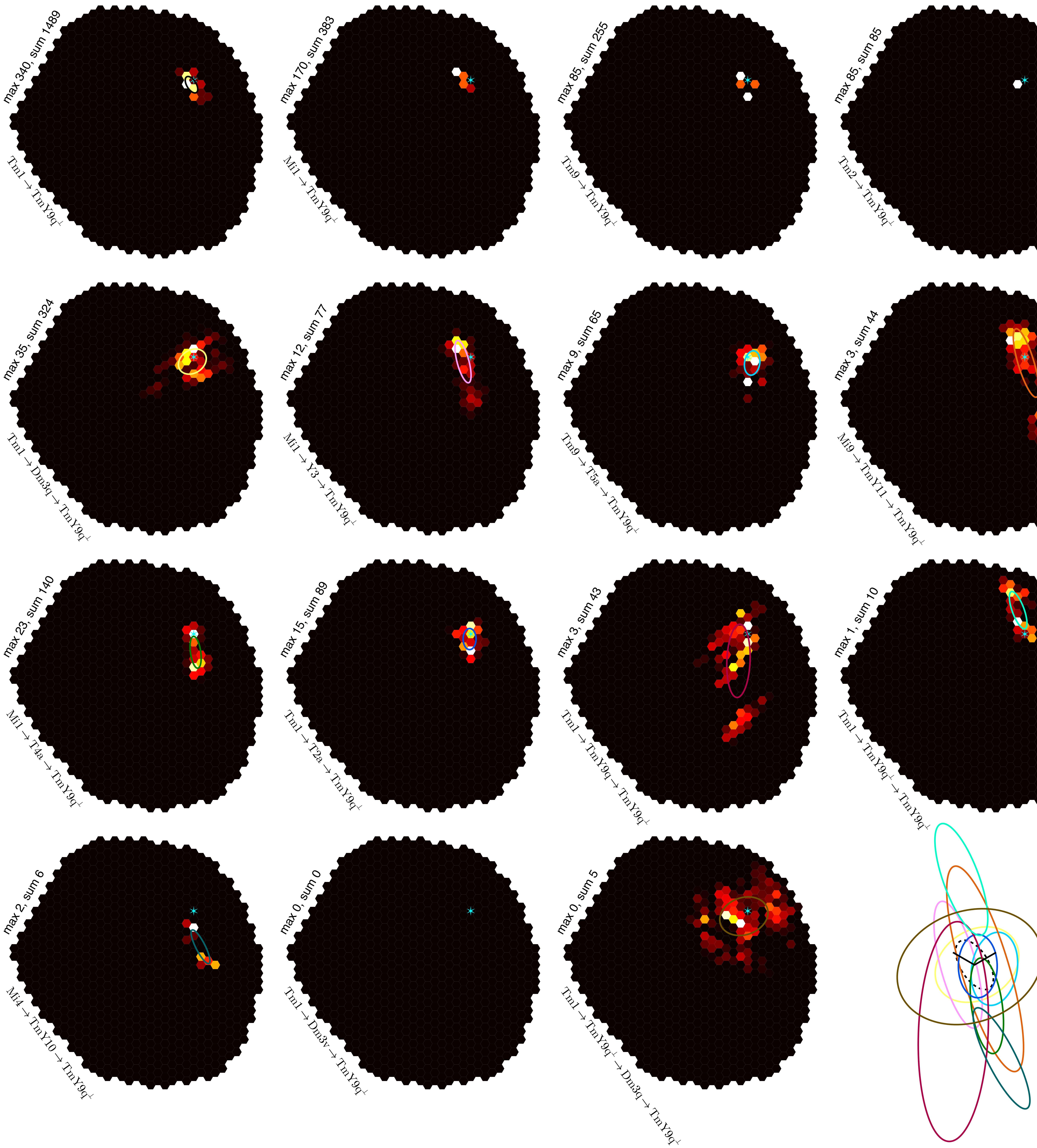
max 340, sum 1489
Tm1 → TmY9q⊥
max 170, sum 383
Mi1 → TmY9q⊥
max 85, sum 255
Tm9 → TmY9q⊥
max 85, sum 85
Tm2 → TmY9q⊥
max 35, sum 324
Tm1 → Dm3q → TmY9q⊥
max 12, sum 77
Mi1 → Y3 → TmY9q⊥
max 9, sum 65
Tm9 → T5a → TmY9q⊥
max 3, sum 44
Mi9 → TmY11 → TmY9q⊥
max 23, sum 140
Mi1 → T4a → TmY9q⊥
max 15, sum 89
Tm1 → T2a → TmY9q⊥
max 3, sum 43
Tm1 → TmY9q → TmY9q⊥
max 1, sum 10
Tm1 → TmY9q⊥ → TmY9q⊥
max 2, sum 6
Mi4 → TmY10 → TmY9q⊥
max 0, sum 0
Tm1 → Dm3v → TmY9q⊥
max 0, sum 5
Tm1 → TmY9q⊥ → Dm3q → TmY9q⊥
